# *Clematis vitalba* Is a Natural Host of the Novel Ilarvirus, Prunus Virus I

**DOI:** 10.3390/v15091964

**Published:** 2023-09-20

**Authors:** Pal Salamon, Zsuzsanna Nagyne-Galbacs, Emese Demian, Adam Achs, Peter Alaxin, Lukáš Predajňa, Evans Duah Agyemang, Francesco Desiderio, Andras Peter Takacs, Wulf Menzel, Dijana Škorić, Miroslav Glasa, Eva Varallyay

**Affiliations:** 1 Applied Plant Genomics Group, Institute of Genetics and Biotechnology, Hungarian University of Agriculture and Life Sciences, Szent-Gyorgyi Albert Street 4, 2100 Godollo, Hungary; salamon.pal@uni-mate.hu; 2 Genomics Research Group, Department of Plant Pathology, Institute of Plant Protection, Hungarian University of Agriculture and Life Sciences, Szent-Gyorgyi Albert Street 4, 2100 Godollo, Hungary; nagyne.galbacs.zsuzsanna@uni-mate.hu (Z.N.-G.); emese.demian@gmail.com (E.D.); desiderio.francesco@uni-mate.hu (F.D.); 3 Institute of Virology, Biomedical Research Center of the Slovak Academy of Sciences, Dúbravská Cesta 9, 84505 Bratislava, Slovakia; adam.achs@savba.sk (A.A.); peter.alaxin@savba.sk (P.A.); lukas.predajna@savba.sk (L.P.); miroslav.glasa@savba.sk (M.G.); 4 Faculty of Natural Sciences, University of Ss. Cyril and Methodius, Nám. J. Herdu 2, 91701 Trnava, Slovakia; 5 Department of Plant Protection, Institute of Plant Protection, Hungarian University of Agriculture and Life Sciences, Deák Ferenc Street 17, 8360 Keszthely, Hungary; evansagyemang932@gmail.com (E.D.A.); takacs.andras.peter@uni-mate.hu (A.P.T.); 6 Plant Virus Department, Leibniz Institute DSMZ-German Collection of Microorganisms and Cell Cultures, Inhoffenstraße 7 B, 38124 Braunschweig, Germany; wulf.menzel@dsmz.de; 7 Department of Biology, Faculty of Science, University of Zagreb, Marulićev trg 9a, 10000 Zagreb, Croatia; dijana.skoric@biol.pmf.hr

**Keywords:** PrVI, *Bromoviridae*, *Clematis*, high-throughput sequencing, alternative host

## Abstract

*Clematis vitalba* L. is a climbing shrub and a pioneer plant in abandoned orchards or vineyards that are widespread in temperate climate zones. In past years, several viruses infecting the *Clematis* species have been identified, including different ilarviruses. Prunus virus I (PrVI) is a recently described ilarvirus, which has been shown to infect sweet cherries and peaches in Greece. Moreover, its presence has been detected in ornamental *Clematis* in Russia. In the present work, we analyzed the virome of wildly growing *C. vitalba* plants from Hungary, Slovakia and Croatia showing different kinds of symptoms using high-throughput sequencing (HTS) of small RNAs or ribodepleted RNAs. Applying HTS enabled us to identify the presence of PrVI in *C. vitalba*, and the bioinformatic analyses were further validated with RT-PCR using PrVI-specific primers and Sanger dideoxy sequencing. Nearly full genome sequences of all three viral RNAs of one Hungarian, two Slovak and one Croatian isolate were determined. Their phylogenetic analysis showed high similarity to each other and to other PrVI isolates described from Central Europe. As the sampled plants were co-infected with other viruses, it is not possible to determine a direct correlation between the infection with PrVI and the observed symptoms. Analyses of different *Prunus* species in stock collection showed infection of several peach and sweet cherry varieties in Hungary. Our results expand the knowledge on the natural host range of PrVI and highlight the necessity to evaluate alternative plant hosts (even non-*Prunus*) of PrVI and the role of the virus in the etiology of the potential diseases.

## 1. Introduction

*Clematis* is a genus of about 380 species belonging to the Ranunculaceae family. They are deciduous, perennial plants growing in temperate regions in the Northern hemisphere and rarely in the tropics. Species and cultivars differing in color and shape of the flowers mainly originate from China and Japan and are used as ornamental plants. They have been constantly bred since 1862, maintained at collections and distributed worldwide, offering more than one hundred different cultivars. The native European version of the genus, *Clematis vitalba* L., is a fast-growing climbing shrub. It grows in and around forested areas, wet, riparian and recently disturbed areas, which is why it is widespread in the vicinity of vineyards and orchards. Although it grows very fast, it never grows as dense mass in this climate. In New Zealand, its invasiveness causes severe damage by blocking the sunlight from the lower vegetation and weighing down the branches of the trees. A biocontrol program to stop its spread was launched in 1989 [1]. To carry out this initiative, a survey examining pathogens affecting *C. vitalba* was completed, revealing that although it can be infected by several fungi and bacteria, only very few of them are pathogenic for this species. *C. vitalba* can also be infected by phytoplasmas [2], mainly those of the 16SrV ribosomal group. In grapevines, this group is associated with a quarantine disease “flavescence dorée” [3]. For this reason, it is considered a weed for vineyards. Although phytoplasma infection sometimes causes yellowing and reddening of the leaves, the infection is usually asymptomatic. *Clematis* species can also be infected with viruses [4]. Eleven different viruses were detected in symptomatic leaves of ornamental *Clematis* species via serological methods and, only recently, using high-throughput sequencing (HTS), an unbiased detection method [5] (Table S1).

Molecular validation and sequence data are only available for tomato bushy stunt virus (TBSV) [5], Moroccan pepper virus (MPV) [6], Clematis chlorotic mottle virus (ClCMV) [5,6,7], cucumber mosaic virus (CMV) [5,8] and Prunus virus I (PrVI) [5] infecting Clematis spp.. Although *Clematis* spp. can be infected with different viruses, *C. vitalba* has only been reported to be infected with ilarviruses: tobacco streak virus (TSV), apple mosaic virus (ApMV) and PrVI (Table 1).

While TSV infection has been reported from the Croatian part of former Yugoslavia [9,13] and from the vicinity of Bologna, Italy, [10] based on serological methods, biotest and electron microscope identification and serological identification of ApMV in Turkey [11] was further validated using RT-PCR [12]. 

PrVI was first described in 2021 from the Imathia region in Greece via HTS analysis of an asymptomatic sweet cherry, Ferrovia cultivar [14]. According to its genome full-length sequence, PrVI belongs to subgroup 1 of the genus *Ilarvirus* (family *Bromoviridae)*. The genome of ilarviruses (genus Ilarvirus in the family *Bromoviridae*) consists of three positive-stranded RNA segments (RNA1–3) [15]. RNA1 is monocistronic and encodes the P1 replicase subunit. RNA2 is monocistronic in the case of subgroups 3 and 4, encoding the P2 replicase subunit, and is bicistronic in subgroup 1 and 2 members, encoding an additional, 2b protein. The putative function of the 2b can be similar to cucumber mosaic virus 2b protein, which plays a role not only in viral movement but also acts as a viral suppressor of silencing [16]. RNA3 is bicistronic, encoding the movement protein (MP) and the coat protein (CP). The CP is expressed from a subgenomic RNA4. Although several ilarviruses are hosted by the *Prunus* species, PrVI is unique because it is the first and still only ilarvirus from subgroup 1 that infects the *Prunus* species [14]. An RT-PCR-based survey in Greece showed that PrVI can also infect peach. Since its original description, PrVI has been reported in Slovenia [17] and Russia [5]. While in Russia, it has been detected in different *Clematis* cultivars (Proteus, Etoile Violette and Ramona in the Nikita Botanical Gardens, Yalta); in Slovenia, it has been found growing in *Picris echioides* L. (Compositae) and sampled as a weed in the surrounding of tomato fields.

In our work, the presence of PrVI was detected in the virome of *C. vitalba* plants in Hungary, Slovakia and Croatia using high-throughput sequencing of small RNAs of the Hungarian samples and ribodepleted RNAs of the Slovak and Croatian samples. Nearly whole genome sequence data (lacking some of the UTR sequences) were used to investigate their phylogenetic relationship. Small-scale surveys testing *C. vitalba* plants and *Prunus* collections were also carried out in order to further investigate the incidence of the virus.

## 2. Materials and Methods

### 2.1. Plant Material and Sample Preparation

#### 2.1.1. Sampling and Small RNA HTS of Hungarian Sample

Leaf samples from different branches of the same symptomatic *C. vitalba* were collected in July 2018 at Budakeszi (close vicinity to Budapest). RNA was extracted from 6 symptomatic leaves (3 showing line patterns and 3 chlorotic spots) using the phenol–chloroform method [18]. Equal amounts of RNA from each leaf were mixed together. The prepared pool was used for small RNA library preparation using the TruSeq Small RNA Library Preparation Kit (Illumina, San Diego, CA, USA), and our modified protocol [19] and were sequenced using a single index on a HiScan2000 using UD GenoMed (Debrecen, Hungary) with 50 bp and a single-end reading. FASTQ files of the sequenced library were deposited to the NCBI SRA database and can be accessed through the accession number PRJNA999171.

Peach and sweet cherry cultivars were sampled in 2017 and 2019, respectively. The peach trees (89 individuals) representing 34 cultivars were kept under an insect proof net and were the same plants whose viromes were characterized recently [20]. The sweet cherry trees (106 trees, representing 32 cultivars) were grown in the same location (Érd, around Budapest) at open field. Four leaves of each tree were collected, and RNA was isolated using CTAB method according to Gambino et al. [21].

#### 2.1.2. Sampling and Total RNA Sequencing of Slovak Samples

Leaf samples of *C. vitalba* were sampled in July 2021 (sample PlCv5) and June 2022 (sample PL622) around Bratislava, Slovakia. Total RNAs from leaves were extracted using a Spectrum^TM^ Plant Total RNA Kit (Sigma-Aldrich, St. Louis, MO, USA). Ribosomal RNA was removed using the Zymo-Seq RiboFree Universal cDNA Kit (Zymo Research, Irvine, CA, USA). Ribosome-depleted RNA preparations were used for double-stranded cDNA synthesis using the SuperScript II kit (Thermo Fisher Scientific, Waltham, MA, USA), and the samples were processed with the transposon-based chemistry library preparation kit (Nextera XT, Illumina, San Diego, CA, USA) followed by HTS on an Illumina MiSeq platform (2 × 150 bp paired reads paired-end sequencing, Illumina, San Diego, CA, USA). 

#### 2.1.3. Sampling and Total RNA Sequencing of Croatian Samples

*C. vitalba* from the vicinity of Zagreb with chlorotic mottling was used in 1989 to inoculate *Chenopodium quinoa* plants as described in Rana et al. [9]. Dried leaves of *Ch. quinoa* with local chlorotic and necrotic symptoms and systemic chlorotic mottling containing Cle-1 isolate of what was then considered to be TSV had been prepared and sent to DSMZ by Davor Miličić in 1991. The isolate was further propagated on *Chenopodium quinoa* and added to the DSMZ plant virus collection under accession No. PV-0309. Extracted total RNA (QIAGEN RNeasy) was sequenced using Illumina NextSeq in 2020, and the genomic sequences were deposited at GenBank (OL584348-50). 

The dried leaves of *Ch. quinoa* containing various Cle isolates from the virus archives of the Department of Biology in Zagreb were used to mechanically inoculate [9] experimental hosts *Nicotiana megalosiphon*, *N. glutinosa, Ch. amaranticolor* and *Ch. quinoa* for the virus revival. Namely, Cle-1 isolate 680 from 1980 and 847 from 1985, as well as Cle-2 isolate 710 from 1981, respectively, were inoculated in 2021. Total nucleic acids (TNAs) were extracted using the CTAB-based method with buffer containing 2% PVP [22]. TNAs were resuspended in 50 microliters of sterile nuclease free water, and an aliquot of 20 microliters was treated with DNase (Promega, Madison, WI, USA) according to the manufacturer’s instructions. After quality checking with a NanoDrop 2000c spectrophotometer (Thermo Scientific, Carlsbad, CA, USA) and Bioanalyzer (Agilent, Santa Clara, CA, USA), the RNA was used for the RT-PCR virus confirmation.

### 2.2. Bioinformatic Analysis of the HTS Data

#### 2.2.1. Pipeline for the Data Evaluation of the sRNA HTS

For bioinformatics analysis of the small RNA reads, we used a CLC Genomic Workbench. After trimming and quality control, longer contigs were built de novo from the non-redundant reads employing an assembler of CLC (de novo assembly) using the default options: a word size of 20, a bubble size of 50 and simple contig sequences with a minimum length of 35 nt (Supplementary Material 1). To diagnose the presence of known viruses, we followed two strategies and used the Qiagen CLC Genomic Workbench: we built longer contigs from the non-redundant reads and BLAST-ed the resulting contigs to the reference genomes of plant-hosted viruses downloaded from GenBank. For each of three RNAs of the PrVI genome, the reads were directly mapped to the reference genome and were counted with and without redundancy (using the map to the reference command allowing one mismatch) (Supplementary Material 1). The number of normalized reads (read/1 million reads: RPM) was then calculated from the mapped redundant reads and the number of total sequenced reads. The coverage (%) of the viral genome was calculated based on a consensus sequence generated from this mapping. We also prepared figures showing the coverage of the genome by sense and antisense virus-specific reads together with the column diagram of the size distribution of virus-derived reads.

#### 2.2.2. Pipeline for the Data Evaluation of the RNAseq

High-quality trimmed reads were used for de novo assembly using a CLC Genomics Workbench v9.5.2 (https://www.qiagenbioinformatics.com/ accessed on 1 September 2023) with automatic graph parameters set and with reads mapped back to contigs with the following parameters (Mismatch cost 2, Insertion cost 3, Deletion cost 3, Length fraction 0.7 and Similarity fraction 0.9) and minimum contig length of 1000 bp. Contigs were aligned to the viral genomes database (ftp://ftp.ncbi.nih.gov/genomes/Viruses/all.fna.tar.gz downloaded 1 July 2023) using CLC Genomics Workbench v9.5.2. Alternatively, the reads were mapped against a selected full-length reference PrVI genome sequence using Geneious v.8.1.9.

### 2.3. Confirmation of the Obtained Results using RT-PCR and Sanger Sequencing

#### 2.3.1. Validation of the sRNA HTS

To validate the results of the bioinformatics analysis, RT-PCR with virus-specific primers were carried out (Table S2). cDNA was synthetized from the RNA representing the prepared small RNA library using random primer and the Maxima H Minus kit (Thermo Fisher Scientific, Waltham, MA, USA), according to the manufacturer’s instructions. The generated cDNA was used as a template for PCR reactions using primers designed according to the small RNA reads. 

For PrVI specific primers, we used Q5 High-Fidelity DNA Polymerase (New England Biolabs, Ipswich, MA, USA). The purified products were cloned into a pJET vector system (Thermo Fisher Scientific, Waltham, MA, USA) and Sanger sequenced using several virus specific primers to cover the whole amplified and cloned viral part. Sequences were deposited into GenBank (Table S3). For surveys, diagnostic primers PrVI-CP-F and PrVI-CP-R amplifying a 351 bp product [14] and Q5 DNA polymerase were used. Amplicons derived from the RT-PCR of pooled samples were directly Sanger sequenced. 

#### 2.3.2. Validation of the RNAseq HTS

Validation of the RNAseq results was performed via two-step RT-PCR using in-house designed PrVI-specific primers (Table S2), spanning each of the five ORFs in all the three viral genomic RNAs. cDNA was prepared from the total RNA using random primers and AMV reverse transcriptase (both from Promega, Madison, WI, USA). The cDNA was used as a template for the conventional PCR performed using GoTaq Green Master Mix (Promega, Madison, WI, USA). The specificity of RT-PCR products was assured by Sanger dideoxy sequencing (Eurofins Genomics, Ebersberg, Germany). 

### 2.4. Serological Test

A standard DAS–ELISA test was carried out (https://www.dsmz.de/fileadmin/_migrated/content_uploads/DAS-ELISA_01 accessed on 1 August 2023) using TSV antiserum AS-0903 available at DSMZ.

### 2.5. Phylogenetical Analysis

Multiple sequence alignments were conducted using ClustalW embedded in MEGA11. Evolutionary history was inferred using the Maximum Likelihood method. The trees were constructed using the best fit model for each alignment, and 500 bootstrap replicates. The trees were drawn to scale, with branch lengths measured in the number of substitutions per site.

### 2.6. Mini-Scale Survey for the Presence of PrVI in Wild Clematis and Hungarian Cherry and Sour Cherry Variety Collection

To survey the presence of PrVI in both *C. vitalba* and different cultivars of cultivated *Prunus* species, leaves of the plants were collected. *C. vitalba* plants growing at different places in the vicinity of Budapest (Budapest, Gödöllő, Szada) showing different leaf chlorosis were sampled in 2018, 2020 and 2021. Total nucleic acid was extracted using the phenol–chloroform method. cDNA was prepared using the Maxima H minus kit (Thermo Fisher Scientific, Waltham, MA, USA). Sweet cherry and peach samples, growing in a cultivar collection at Érd, were also tested for the presence of PrVI. From these plants RNA was extracted with the CTAB method [21]. cDNA from these samples was prepared using the Revertaid First Strand kit (Thermo Fisher Scientific, Waltham, MA, USA), using random primers. RT-PCR was carried out using Q5 DNA polymerase (New England Biolabs, Ipswich, MA, USA) and previously published PrVI-CP-F/PrVI-CP-R primers [14]. 

### 2.7. Mini-Scale Survey for the Presence of PrVI in Wild Clematis in Slovakia

To survey the presence of PrVI in wild *Clematis vitalba* in Slovakia, leaf samples of five plants growing in different locations of Bratislava was harvested and analyzed with two-step RT-PCR as described in Section 2.3.2 using PVIc-1MTR_F/PVIc-1MTR_R primers (Table S2). Amplification products were further validated with the Sanger dideoxy sequencing.

## 3. Results

### 3.1. C. vitalba in Hungary Was Found Infected with PrVI

**Figure 1:**
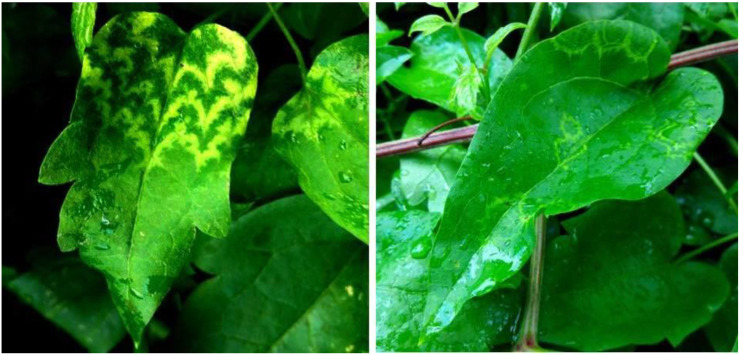
Leaves from a symptomatic *Clematis vitalba* plant showing bright yellow line pattern and ringspot symptoms collected in July 2018 at Budakeszi (close vicinity to Budapest, Hungary) (photo: Pal Salamon).

*C. vitalba* growing at the edge of a woodland area in Budakeszi (vicinity of Budapest, Hungary) showed a nicely shaped line pattern symptom, suggesting a potential virus infection (Figure 1).

**Figure 2:**
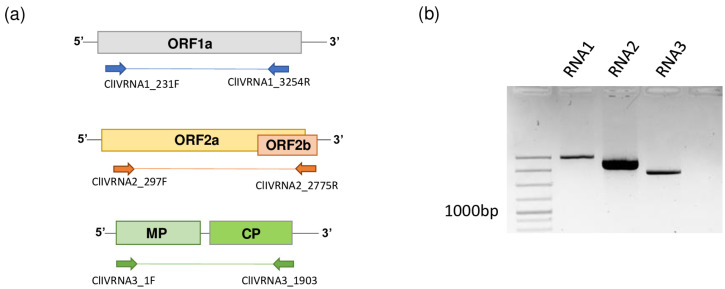
Validation of the small RNA HTS. (**a**) Primers were designed based on the *Ilarvirus*-genus-specific contig sequences able to amplify most of the each of three genomic RNAs. (**b**) Agarose gel electrophoresis of the RT-PCR productsobtainedwith the primers shown in (**a**). M is the GeneRuler 100 bp Plus DNA ladder (Thermo Fisher Scientific, Waltham, MA, USA).

Mapping of the contigs built up from the sequenced small RNA reads resulted in hits for several different ilarviruses belonging to subgroup 1 (Supplementary Material 1). This pattern suggested the presence of a distinct ilarvirus whose reference has not been included in the NCBI GenBank Reference list. Based on the ilarvirus mapped contig sequences, primers were designed for each of three RNAs (Figure 2a, Table S2). RT-PCR using these primers targeting the three different putative viral RNAs amplified products at the expected size (Figure 2b). 

After the cloning and sequencing of these products, BLAST search of their sequences showed high similarity to PrVI, a virus described in 2021 [14]. With this knowledge, the bioinformatic analysis was repeated including genomes of PrVI. This time 571,488 trimmed reads and 2195 contigs could be mapped to either segment of the PrVI genome, covering more than 96% of the viral genomes (lacking part of the 5′ and 3′ UTR) (Supplementary Material 1, Table S3).

Size distribution of the PrVI mapped reads showed high over-representation of 21–22 nt long reads (Supplementary Material 1), indicating a strong antiviral silencing host response. Sequences of the cloned viral segments of this CleHU variant were deposited into the NCBI GenBank (acc. nos. RNA1: OR192162, RNA2: OR192163 and RNA3: OR192164).

### 3.2. C. vitalba in Slovakia Is Infected with PrVI

**Figure 3:**
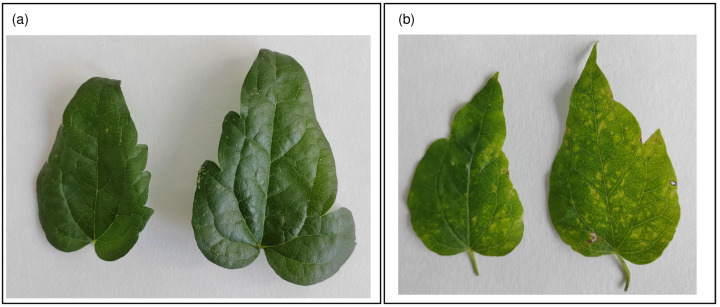
*C. vitalba* plants from Slovakia showing mosaic like symptoms hosting (**a**) PL622 and (**b**) PlCv5 PrVI isolates.

As a part of viral monitoring in the agro-ecological interface in Slovakia, we have sampled two *C. vitalba* plants, showing virus-like symptoms: mosaic in the case of PlCv5 and slight leaf deformations and puckering for PL622 (Figure 3).

Leaf samples from the symptomatic plants were collected and served as a material for ribodepleted RNAseq. Bioinformatic analysis of the sequenced reads identified CMV in PlCv5 (not discussed further in this study) and PrVI in two clematis samples (PlCv5 and PL622) (Table 2), from which the nearly complete PrVI genome sequences were obtained (lacking part of the 5′ and 3′ UTR) (Table S3) and deposited into GenBank (PlCv5 RNA1: OR452176, RNA2: OR452178 and RNA3: OR452180; PL622 RNA1: OR452175, RNA2: OR452177 and RNA3: OR452179).

To obtain a genome-wide confirmation of the presence of PrVI, ORFs located on all three genomic RNA segments were specifically targeted using RT-PCR (Figure S1a). 

In the PlCv5, amplicons representing the coding part for methyltransferase, helicase, a gap region between the two, RNA-dependent RNA polymerase (RdRp), movement protein (MP) and CP were successfully amplified (Figure S1b), while the validation of the amplicon representing the product of the 2b ORF was not successful with the primers designed based on the HTS data and similarities of known full-lenght PrVI genomes.

### 3.3. PrVI Presence from Archival Croatian Cle Isolates

*C. vitalba* was found to be a host of a new strain of TSV in the 1980s [9]. The inoculated experimental plant materials, mostly *Ch. quinoa* leaves, were dried and deposited as Cle-isolates in the virus archives of the Department of Biology in Zagreb. Essentially, two types of isolates named Cle-1 and Cle-2 were deposited over the 1980s. In the Cle-1 isolates, the symptomatology in the original host was characterized by chlorotic spots and in the Cle-2 by yellow netting [13]. Both isolates originated from clematis plants in the vicinity of Zagreb. We reconstructed from laboratory notes that the last isolate of Cle-1 stored prior to this research (no. 925 from 1989) was received at DSMZ in 1991 and used to generate the knownPrVI RNA1-3 full-lenght sequence records (GenBank acc. no. OL584348-50).

**Figure 4:**
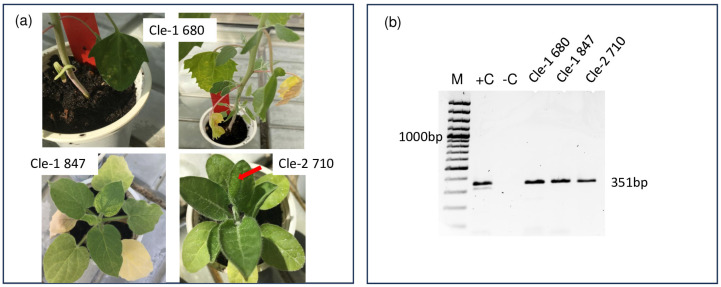
Biotest of the Croatian Cle-1 isolate. (**a**) Symptoms in experimental plants after inoculation with the Croatian archival Cle isolates of PrVI: local chlorotic mottling 8 dpi in *Chenopodium quinoa* with Cle-1 isolate 680 (**top left**). The same plant 30 dpi with systemic mottling (**top right**). Systemic yellow net in *Nicotiana glutinosa* top leaves infected with Cle-1 isolate 847 16 dpi (**bottom left**) and *N. megalosiphon* with Cle-2 710 with less intense netting 27 dpi (red arrow). (**b**) RT-PCR confirmation of the PrVI infection in the sampled test plants using diagnostic primers: PrVI_CP-F and PrVI_CP-R amplifying 351 bp of the coat protein encoded on RNA3. M is the GeneRuler 100 bp Plus DNA ladder (Thermo Fisher Scientific, Waltham, MA, USA).

The PV-0309 isolate was tested with DAS–ELISA with the TSV antiserum AS-0903 available at DSMZ, which was raised against a TSV isolate originating from a *Vicia faba* sample from Sudan (PV-0903). PV-0309 showed no positive reaction (OD < 1.5 no multiplication sign is needed OD of the healthy controls), whereas the homologous TSV isolate PV-0903 and PV-0615 (originating from sunflower/India) showed a strong reaction (OD > 8 no multiplication sign is needed O OD of the healthy controls), indicating a serological differentiability. Out of four archival Cle-isolates mechanically inoculated here, three produced symptoms in *Ch. quinoa* and *Nicotiana* plants and thus were successfully revived. Cle-1 680 from 1980 and 847 from 1985 expectedly [9] showed local chlorotic spots on *Ch. quinoa,* turning into necroses later during the course of infection, and systemic mottling. Yellow net appeared in *N. megalosiphon* and *N. glutinosa* in Cle-1 isolates, whilst Cle-2 isolate 710 from 1981 produced only yellow net in *N. megalosiphon* (Figure 4). 

The subsequent RT-PCR experiments with PrVI primers [14] confirmed that nucleic acid extracts from the isolates Cle-1 680 and 847, as well as Cle-2 isolate 710, were also the source of specific amplification products.

### 3.4. Sequence Variability and Phylogenetic Analysis of the PrVI Strains Present in C. vitalba

Sequence comparison of the PrVI variants showed that they are very homologue. Identity of nucleotides are 96.73–99.194%, 96.352–98.891% and 94.19–98.849% for RNA1, RNA2 and RNA3, respectively (Table 3). 

**Figure 5:**
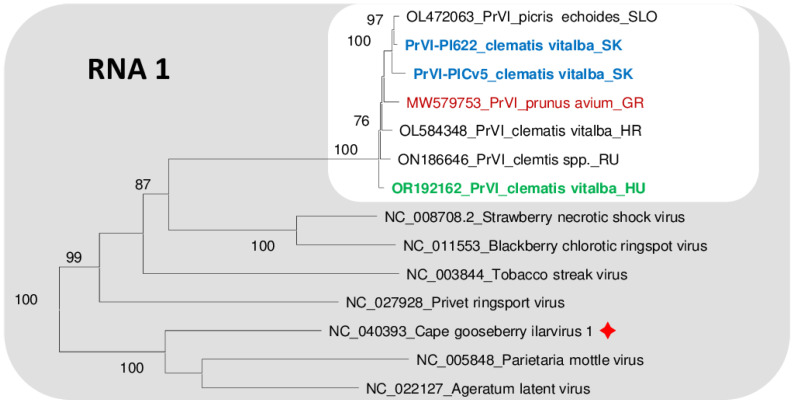
Phylogenetic analysis of the nucleic acid sequences of RNA1 of PrVI and other ilarviruses belonging to group 1. Viruses are referred by the GenBank accession numbers of the reference genomes, following by the full name of the virus. In case of PrVI only the abbreviated PrVI is displayed followed by the host name and the origin of the country (SLO—Slovenia, SK—Slovakia, GR—Greece, HR—Croatia, RU—Russia and HU—Hungary). The red sign indicates a new virus with a reference of which sequence was not included in the phylogenetical analysis in the original description of the virus [14].

**Figure 6:**
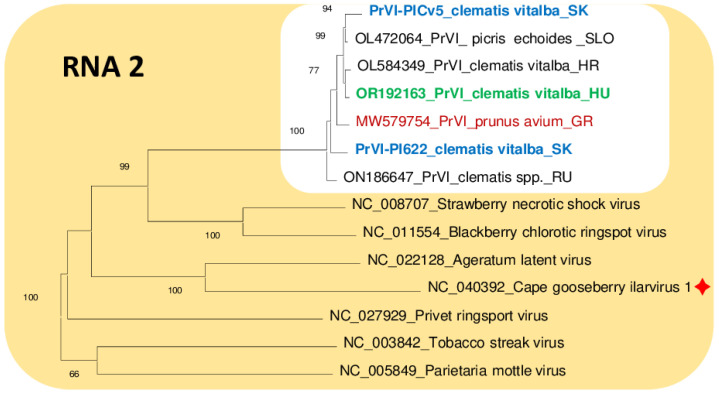
Phylogenetic analysis of the nucleic acid sequences of RNA2 of PrVI and other ilarviruses belonging to group 1. Viruses are referred by the GenBank accession numbers of the reference genomes, following by the full name of the virus. In case of PrVI only the abbreviated PrVI is displayed followed by the host name and the origin of the country (SLO—Slovenia, SK—Slovakia, GR—Greece, HR—Croatia, RU—Russia and HU—Hungary). The red sign indicates a new virus with a reference of which sequence was not included in the phylogenetical analysis in the original description of the virus [14].

**Figure 7:**
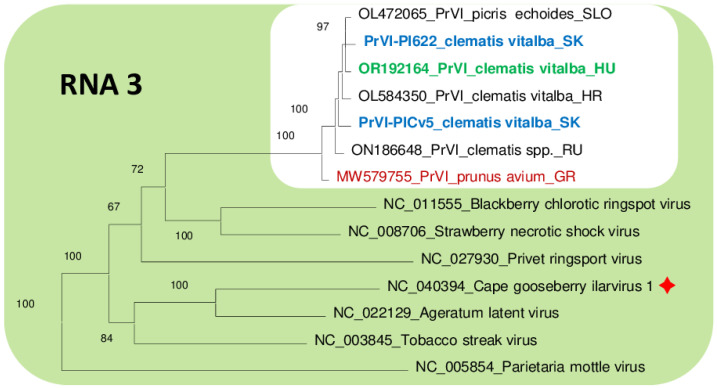
Phylogenetic analysis of the nucleic acid sequences of RNA3 of PrVI and other ilarviruses belonging to group 1. Viruses are referred to by the GenBank accession numbers of the reference genomes, following by the full name of the virus. In case of PrVI only the abbreviated PrVI is displayed followed by the host name and the origin of the country (SLO—Slovenia, SK—Slovakia, GR—Greece, HR—Croatia, RU—Russia and HU—Hungary). The red sign indicates a new virus with a reference of which sequence was not included in the phylogenetical analysis in the original description of the virus [14].

While the Slovak variants showed the closest identity to the Slovenian variant sequenced from *Picris echiodies*, the three RNAs of the Hungarian variant showed the highest identity to either Croatian, Slovenian or the Slovak variants. The Croatian variant showed the highest identity to the Hungarian Cle isolate. This close relationship and clustering are also shown in the phylogenetic trees prepared for either RNA1, RNA2 or RNA3 sequences (Figure 5, Figure 6 and Figure 7). The most variable sequence was found for the MP and CP encoded by RNA3. On the phylogenetic tree investigation relationship of RNA3 Slovak, Hungarian, Croatian and Slovenian isolates cluster together, a bit distantly from the Greek and Russian variants (Figure 5, Figure 6 and Figure 7).

Investigation of the proteins encoded using the PrVI variants showed that RNA1-encoded proteins have higher than 98.4% identity, RNA2-encoded RdRp proteins have higher than 98.2% identity and RNA3-encoded MP protein have more than 98% identity between different strains, while amino acid identity between the RNA2-encoded 2b and RNA3-encoded CP is more variable, higher than 94.1 and 95.6%, respectively (Table S4). The identity among the *C. vitalba* isolates (indicated by green) were usually higher than the identity of isolates infecting other plant species (indicated with different colors).

### 3.5. Survey of PrVI in Symptomatic C. vitalba and Prunus sp. Stock Collection

**Figure 8:**
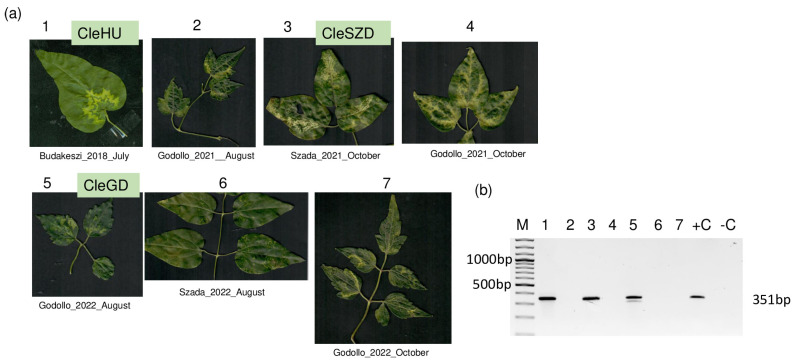
Clematis mini survey. (**a**) Symptoms of the sampled leaves of *C. vitalba*, indicating the geographical origin and the year and month of sampling. (**b**) Result of the RT-PCR test of the sampled *C. vitalba* plants using diagnostic primers: PrVI_CP-F and PrVI_CP-R [14] amplifying 351 bp part of the coat protein encoded on RNA3. M is the GeneRuler 100 bp Plus DNA ladder (Thermo Fisher Scientific, Waltham, MA, USA). Sequences of the amplified viral RNA3 segments were directly sequenced (GenBank acc. numbers are CleSZD–OR485309 and CleGD–OR485310).

A mini survey of seven symptomatic *C. vitalba* plants in the close vicinity of Budapest (Szada and Gödöllő) showed that some of them are infected with PrVI, but the presence of PrVI was not connected to the presence of the symptoms (Figure 8).

Moreover, we further tested five *C. vitalba* leaf samples collected in Bratislava (Slovakia). Only one of these samples tested positive for the presence of PrVI, confirming there is no direct correlation between PrVI infection and the observed symptom manifestation.

In order to investigate the presence of PrVI in different *Prunus* species in Hungary, we surveyed 89 peach and 106 sweet cherry trees at Erd, in a stock collection in a close vicinity of Budapest, representing 34 and 32 cultivars, respectively. All individuals of ten peach (Fantasia, Mariska, Harko, Regina, Champion, Suncrest, Öb166/1, Genadix 4, Elvira and Starling) and three sweet cherry cultivars (Valerij cskalov, Szomolyai fekete and Aida) were found infected with PrVI, suggesting that the origin of the infection was the clonal propagation of the cultivar (Table S5). 

## 4. Discussion

As a part of a virus monitoring in wildly growing woody and perennial plants, we have sampled symptomatic *C. vitalba* plants showing virus-like symptoms both in Hungary and Slovakia. HTS of the samples identified the presence of PrVI, which could be confirmed using RT-PCR.

Our bioinformatic pipeline for small RNA HTS using BLAST search employed by NCBI GenBank Viral Reference Genomes as a database failed to detect the presence of the recently described virus (PrVI), having no reference in GenBank. Although we were not capable of its direct identification, the results showed hits to several different ilarviruses, suggesting the infection with a virus belonging to this genus but lacking a reference genome. This result could be validated, and during the validation, the identification of the virus was possible, showing that small RNA HTS is a potent diagnostic tool. Size distribution of the virus-derived siRNAs showed extreme bias of 21–22 nt long reads. This size range is typical of the products of antiviral DICERs: DCL4 and DCL2 [23]. The high number of the PrVI-derived siRNAs suggests that it induces a strong host defense response. This response can be weakened in long plant–virus coexistence, as it was the case in the grapevine rupestris stem-pitting associated virus infected grapevine [24], making sRNA HTS unable to detect its infection [25]. Recently described viruses, like grapevine virus T (GVT) are often thought to be overlooked because of their latency and absence of strong host response [26]. This seems not to be the case in PrVI. Our mini-scale survey of *C. vitalba* plants showed that plants with different virus-like symptoms are either infected with PrVI or not, suggesting no direct correlation of the symptom manifestation and the presence of this virus. In the infected peach and sweet cherry trees, no specific symptoms were observed. Furthermore, we cannot correlate the presence of the symptoms on *C. vitalba* with the infection of PrVI either, as there were other viruses present in these plants; nonetheless, at least for the *C. vitalba,* we saw a very strong RNAi response. Moreover, because we have intentionally selected these specific plants based on their symptomatology without the previous knowledge of the PrVI mixed co-infection, a survey for the presence of the PrVI in asymptomatic *C. vitalba* environmental samples is needed in order to correctly explain the disease etiology and the contribution of PrVI to the symptomatology observed. 

Viromes of *Clematis* plants have been mainly investigated using serological methods in the past. Although the infection of *C. vitalba* has been reported by TSV and ApMV, no sequence data are available for these strains. TSV was identified using serological methods [9,10], while for ApMV, an RT-PCR test was also presented [12]. Cle strain of TSV reacted with antisera against the North American TSV strain or showed a very faint cross reaction with the other TSV antisera suggesting its distant relationship among TSV [9]. It was found to be different from other TSV strains in its host range and symptoms induced in some common host, raising the possibility of the presence of another ilarvirus related to TSV. The sequence record from *Clematis* in Croatia, near Zagreb, showed that the archival material from 1991 used for obtaining this sequence (DSMZ PV-0309 strain, having only GenBank record) had been indeed infected by PrVI. This isolate also shows no serological reaction with the DSMZ TSV antiserum (AS-0903) in DAS–ELISA, indicating serological differentiability based on differences in the antigenic domains of the coat protein. The course of events reconstructed in this research showed that this sequence comes from the so-called Cle-1 isolates stored in the virus archive of the Department of Biology in Zagreb. Additional RT-PCR analyses of two Cle-1 and one Cle-2 isolates from the 1980s confirmed the presence of PrVI amplicons in the archival *Ch. quinoa* dried leaves from Zagreb (Figure 4b). Amazingly, these isolates showed infectivity in biological tests after four decades in storage (Figure 4a). It is therefore quite likely that this original TSV infection in *Clematis* reported from former Yugoslavia could have been falsely diagnosed due to the lack of knowledge about PrVI. TBLASTX search of the RNA sequence of PrVI DSMZ PV-0309 strain (OL584350) showed high identity (about 80%) in the amino acid sequence of the MP encoded by TSV dahlia strain [27] or with a TSV strain infecting summer squash in Georgia, USA [28]. The same amino acid identity (80%) was also found in the case of corresponding RNA1- and RNA2-encoded proteins. The presented evidence strongly suggests that the TSV report in *Clematis* from today’s Croatia [9] was the earliest detection of PrVI. TSV has also been reported from *C. vitalba* in Italy [10]. With our current knowledge we can also question if it was really TSV, but in the absence of the original plant material we cannot prove or rule out this statement. In contrast, even in the lack of the sequence of the ApMV strain found in *Clematis*, this infection cannot be a falsely diagnosed PrVI as the amino acid identity of the Greek sweet cherry strain and the ApMV from *Clematis* in Turkey is low. Moreover, the primers that were used to amplify the partial RNA3 of ApMV could not anneal to the PrVI strains.

PrVI has been described from *Prunus* spp. in Greece [14], but later, its presence was dominantly described from *Clematis* spp. Whether it is a real bias or just a result of random and uneven sampling of the rural samples is a question that needs to be investigated later. Nonetheless, *Clematis* spp. could act as a potent natural PrVI host (in addition to *Prunus* spp.), thus making it quite an interesting target of epidemiological studies regarding the spread or persistence of this new ilarvirus species in the environment.

Chirkov et al. [5] observed that the 2b and MP proteins of the sweet cherry PrVI isolate (MW579754 and MW579755, respectively) differ the most in their nucleotide as well as amino acid sequences from the clematis PrVI isolates. They hypothesize that the non-synonymous mutations seem to be responsible for these changes, which could reflect a different host adaptation of this virus species due to the role of these proteins in the cell-to-cell viral transport. This pattern seems to be perpetuated in the case of our samples as well. For example, regarding the phylogenetic trees, no host specificity can be strictly determined from the clustering of the RNA1 and RNA2 (probably due to the relatively shorter length of 2b in comparison to RdRP) segments (Figure 5 and Figure 6). However, some host-related pattern is visible in the case of the RNA3 segment encoding the MP, and although the relative distances of the branches between the different PrVI isolates are still short (on the level of the same species), they also seem to be a bit more clearly separated from each other (Figure 7). This is also supported by the fact that the sweet cherry PrVI isolate is the most diverted from other PrVI isolates reported in this work in the case of the RNA3 segment (94–95%, Table 3)—in comparison, RNA1 and 2 segments show divergence of only 96–97%, which could be explained by the fact that replicase and polymerase ORFs are responsible for the essential (host-independent) viral replication processes, thus these sequences must stay unchanged during the evolutionary pressure. Interestingly, regarding this MP host-sequential adaptability, it is worth to mention that the MP of the clematis PrVI isolate from Croatia (OL584350) shows high identity on the level of amino acid sequence with the MP of the TSV (which is in the same species cluster as PrVI, Figure 5, Figure 6 and Figure 7) isolate from dhalia (LC030107) and summer squash (MK307506) (Figure S2). Interestingly, the highest (but still very low) variability could be detected for 2b protein, a potential viral silencing suppressor, which also needs to be adapted to the host.

Our small-scale survey of *Prunus* species identified the infection of several peach and sweet cherry cultivars in a small stock collection in Hungary. The peach trees, as potential plum pox virus (sharka) hosts, were kept under an insect proof net, while the sweet cherry trees were grown in an open field. We found 38% of the peach and 30% of the sweet cherry trees tested positive for PrVI, showing that similarly to Greece, the virus is present in the cultivated *Prunus* species in Hungary. The PrVI infection was restricted to some cultivars, and we could not detect the infection between neighboring different cultivars, suggesting that the infection in this case happened through propagation. *Prunus* species infected with the virus did not show any specific symptoms, while symptoms in *Clematis* could not be directly linked to the presence of the virus, as the sampled plants were co-infected with other viruses.

PrVI has also been described from an herbaceous plant *Picris* sp. in Slovenia, suggesting a wider host range of the virus than just *Prunus* or *Clematis* genera members. 

As an ilarvirus, it is highly possible that PrVI can be transmitted by pollen [16,29]. Moreover, the presence of Cle-1 isolates from Zagreb was proven in the pollen of experimentally inoculated *Ch. quinoa* plants [9]. The infection of *Clematis* could thus be acquired by pollen, and the same may be true in other cultivated or wild plants. Further research is needed to clarify these epidemiological scenarios. 

Our finding expands *Clematis* host range reported previously and suggests *Clematis vitalba* could be a natural reservoir of PrVI. However, the epidemiology of the virus, having apparently a broad host range, remains still to be elucidated. These data also highlight the necessity to reconsider alternative plant hosts of PrVI and their role in the etiology of the potential diseases.

We do not know if PrVI can be harmful on the cultivated *Prunus* trees and cannot suggest at this stage a directive for its possible regulation. This question could be answered only after further research investigating the biology, symptomatology and transmission of PrVI in detail. Based on further information on how widespread PrVI is and if the virus has a negative impact on cultivated plants, decisions can be made regarding a necessary regulation or consideration in the certification of planting material.

## 5. Conclusions and Dedication

Before the rapid development of nucleic-acid-based research, plant virology relied mainly on biological characterization including symptom description, transmission experiments as well as serological and electron microscopical detection of viruses. The huge work of classical virologists enabled us to find correlations between viral diseases and sequence data of the later sequenced viruses. With the fast evolution of sequencing techniques, an unexpected number of plant-infecting viruses are currently being described, and their presence can easily be physically verified. Classical virologists took care of the virus isolates they characterized and deposited them in viral collections. Our research shows how recent and classical virus research can complement each other to explore and clarify the legacy of these great classical virologists—represented in our case by Professors Davor Miličić and Giovanni Paolo Martelli. We would like to honor their tremendous work and dedicate our research to them.

## Figures and Tables

**Table 1:** Ilarviruses reported to infect *Clematis* spp.

Virus	Host Plant	Country of Origin	Symptoms	Method for the Detection	Sequence Data (GenBank Accession Number)	Reference
TSV	*C. vitalba*	Yugoslavia (surroundings of Zagreb and Poreč)	chlorotic spots, yellow netting and lack of symptoms	biotest, electron microscopy (EM), serology	no sequence record	[9]
	*C. vitalba*	Italy (around Bologna)	yellow mosaic and vein yellowing	biotest, EM, serology	no sequence record	[10]
ApMV	*C. vitalba*	Turkey	lack of symptoms	biotest, ELISA	no sequence record	[11,12]
				RT-PCR	no sequence record	
PrVI	3 *Clematis* cultivars: Etoile Violette, Proteus and Ramona	Russia	multiple infections, not possible to link to PrVI	HTS and RT-PCR	complete genome: ON186646-48	[5]
	*C. vitalba*	Hungary (around Budapest)	line pattern and chlorotic spots	small RNA HTS, RT-PCR	almost complete genome: OR192162-64	this work
	*C. vitalba*	Slovakia (around Bratislava)	mosaics, slight leaf deformation and puckering	RNAseq HTS, RT-PCR	almost complete genome: OR452175-80	this work
	*C. vitalba*	Croatia (around Zagreb)	no symptom mentioned in the record	RNAseq HTS	complete genome: OL584348-50	unpublished, only GenBank entry

**Table 2:** HTS statistics for Slovak samples.

	Total Number of Reads	PrVI Ref		Number of Assembled Reads	Mean Length of Reads Mapping (bp)	Sequence Depth Mean Coverage	Coverage of the Viral Genome (%)
PICv5	2,875,002	MW579753	RNA 1	3018	92.3	79.8	97.50%
		MW579754	RNA 2	1634	89.3	49.9	97.00%
		MW579755	RNA 3	1608	88.4	60.9	98.80%
PL622	4,847,048	MW579753	RNA 1	2807	148.3	105.6	100%
		MW579754	RNA 2	1956	155.6	95.4	100%
		MW579755	RNA 3	1896	138	103.3	100%

**Table 3:** Percent identity matrixes of the nucleotide sequences of the PrVI RNAs.

RNA1	OR452176 PlCv5_SK	OR452175 Pl622_SK	OR192162 CleHU_HU	OL472063_ Pichris_SLO	ON186646 Cle_RU	MW57975 c18_GR	OL584348 PV0309_CR
OR452176_PlCv5_SK	x	98.567	97.651	98.478	97.164	97.045	97.493
OR452175_Pl622_SK	98.567	x	98.147	99.194	97.612	97.493	98.06
OR192162_CleHU_HU	97.651	98.147	x	98.114	98.279	97.617	98.61
OL472063_Pichris_SLO	98.478	99.194	98.114	x	96.877	96.732	97.458
ON186646_Cle_RU	97.164	97.612	98.279	96.877	x	97.513	97.747
MW57975_c18_GR	97.045	97.493	97.617	96.732	97.513	x	97.183
OL584348_PV0309_CR	97.493	98.06	98.61	97.458	97.747	97.183	x
RNA2	OR452178 PlCv5_SK	OR452177 Pl622_SK	OR192163 CleHU_HU	ON186647 Clem_RU	OL472064 Picris_SLO	OL584349 PV0309_CR	MW579754 c18_GR
OR452178_PlCv5_SK	x	96.459	98.514	96.352	98.891	98.355	97.532
OR452177_Pl622_SK	96.459	x	96.627	96.795	96.936	96.689	96.865
OR192163_CleHU_HU	98.514	96.627	x	96.867	98.916	98.916	97.952
ON186647_Clem_RU	96.352	96.795	96.867	x	96.745	96.641	97.298
OL472064_Picris_SLO	98.891	96.936	98.916	96.745	x	98.687	97.549
OL584349_PV0309_CR	98.355	96.689	98.916	96.641	98.687	x	97.321
MW579754_c18_GR	97.532	96.865	97.952	97.298	97.549	97.321	x
RNA3	OR452180 PlCv5_SK	OR452179 Pl622_SK	OR192164 CleHU_HU	ON186648 Clem_RU	OL584350 PV0309_CR	OL472065 Picris_SLO	MW579755 c18_GR
OR452180_PlCv5_SK	x	97.848	97.803	96.811	97.619	97.893	95.446
OR452179_Pl622_SK	97.848	x	98.849	95.77	97.015	97.43	94.197
OR192164_CleHU_HU	97.803	98.849	x	97.503	98.08	98.697	96.058
ON186648_Clem_RU	96.811	95.77	97.503	x	96.465	96.154	96.181
OL584350_PV0309_CR	97.619	97.015	98.08	96.465	x	97.527	95.27
OL472065_Picris_SLO	97.893	97.43	98.697	96.154	97.527	x	94.68
MW579755_c18_GR	95.446	94.197	96.058	96.181	95.27	94.68	x

## Data Availability

Raw files of sRNA and RNAseq HTS can be accessed at SRA BioProject PRJNA999171. GenBank accession numbers of the PrVI stains related to this report can be accessed at NCBI GeneBank: OR192162-64, OR452175-80, OL584348-50 and OR485309-10.

## Supplementary Materials

The following supporting information can be downloaded at https://www.mdpi.com/article/10.3390/v15091964/s1, Supplementary Material 1: bioinformatic analysis of the small RNA HTS; Supplementary Figures: Figure S1: validation of RNA sequencing; Figure S2: alignment of the amino-acid sequences of the PV-0309 PrVI isolate to closely related TSV isolates; Supplementary Tables: Table S1: viruses has been reported to infect Clematis species; Table S2: sequences of the PCR primers used for virus detection with their appropriate references; Table S3: GenBank identifiers of the amplified and sequenced virus specific products; Table S4: percent identity matrix of the amino acid identity of the proteins encoded by different PrVI strains; Table S5: result of the survey testing the presence of PrVI in different cultivars of peach and sweet cherry in Hungary.
