# Supplementary material for: Clematis vitalba Is a Natural Host of the Novel Ilarvirus, Prunus Virus I
Source: Viruses. 2023 Sep 20;15(9):1964. doi: 10.3390/v15091964 (PMC10536899; doi:10.3390/v15091964)
Supplement: Supplementary file 1 [file viruses-15-01964-s001.zip › Salamon et al_Supplementary Figures.pdf]

viruses

Supplementary Figures to

Clematis vitalba is a natural host of a novel ilarvirus, Prunus virus I

Pal Salamon[1], Zsuzsanna Nagyné-Galbács[2], Emese Demian[2], Adam Achs[3], Peter Alaxin[3,4], Lukáš Predajňa[3], Evans Duah Agyemang[5], Francesco Desiderio[2], Andras Takacs[5], Wulf Menzel[6], Dijana Škorić[7], Miroslav Glasa[3,4], Éva Varallyay[2*]

*Correspondence: varallyay.eva@uni-mate.hu

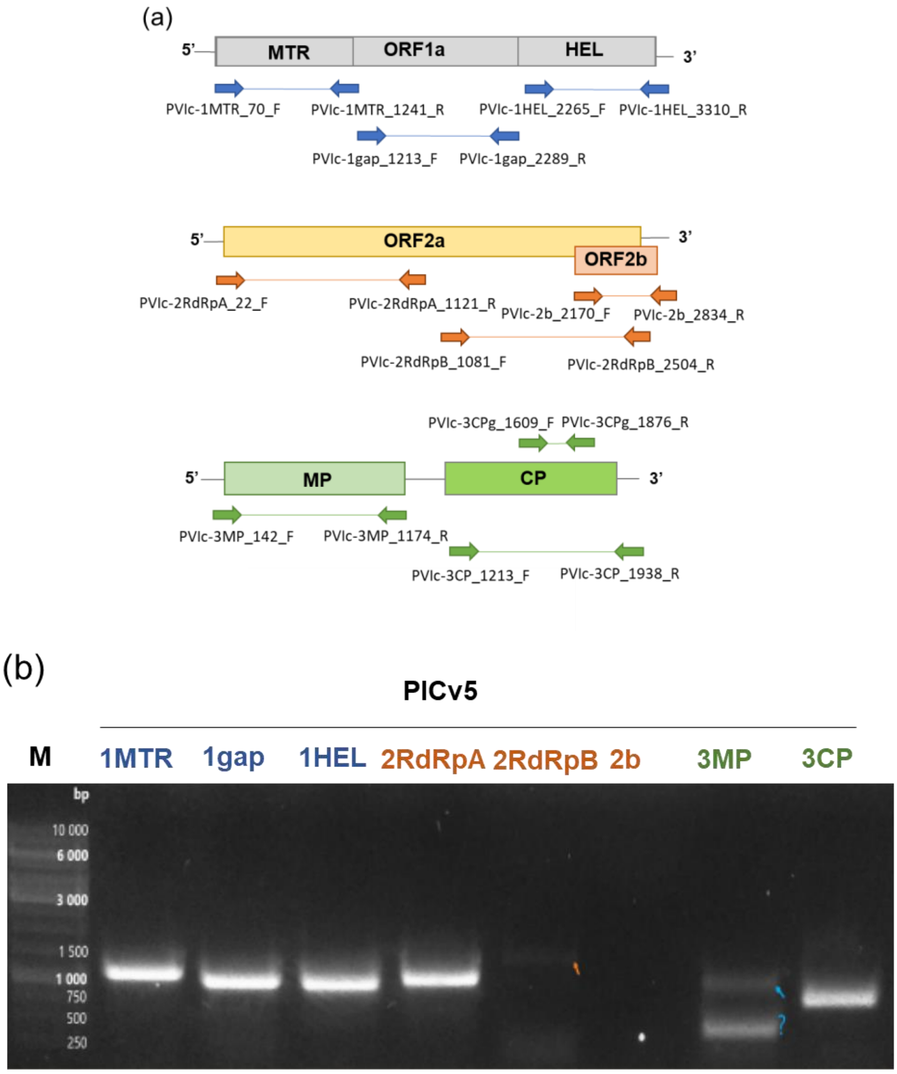

Figure S1. Validation of RNA sequencing. (a) Primers were designed based on the ilarvirus specific contig sequences able to amplify almost all three RNAs of the entire genome. (b) result of the RT-PCR. M corresponds to GeneRuler 1kb DNA Ladder (Thermo Fisher Scientific, Waltham, MA, USA). Arrows indicate the positive products.

Tobacco streak virus gene for movement protein, complete cds, isolate: dahlia
Sequence ID: LC030107.1Length: 873Number of Matches: 11
Range 1: 1 to 753GenBank Next Match Previous Match

Alignment statistics for match #1

Score	Expect	Identities	Positives	Gaps	Frame
502 bits(1091)	7e-143	203/251(81%)	234/251(93%)	0/251(0%)	+3/+1

```
Query  210   MALTPSYRALTFSADDESSLEKSVSDALSGCVELNMGLRRCAAFPATNTEAFLCELTTKE  389
             MAL P+ +ALTFSA+DE+SLEK++++ALSG VE+NMGLRRCAAFPA NT AFLCELTTKE
Sbjct  1     MALVPTMKALTFSAEDETSLEKAITEALSGSVEINMGLRRCAAFPAVNTGAFLCELTTKE  180

Query  390   TKTILGKFADKVRGRVFVDHAVIHMMYIPVILSTTHAVAELKIRNMATGDELYGGTKVNL  569
             TK+ +GKF+DKVRGRVFVDHAVIH+MYIPVIL+TTHA+AELK++N+ATGDELYGGTKVNL
Sbjct  181   TKSFIGKFSDKVRGRVFVDHAVIHLMYIPVILNTTHAIAELKLKNLATGDELYGGTKVNL  360

Query  570   NEAFILTMTWPRSLFADAVHAHKGLYLGGTVNCATSVPKGCNIGMWYPMWSEKVSNKQLY  749
             ++AFILTMTWPRSLFA+AVH+H+GLYLGGTV+CA+SVP    IGMWYPMWSEKVSNKQLY
Sbjct  361   SKAFILTMTWPRSLFAEAVHSHRGLYLGGTVSCASSVPANAKIGMWYPMWSEKVSNKQLY  540

Query  750   QNTTNIVNTKALETFTRTMISSDREMRSLLRSRASIDIAAKTAEKPVLCSSHVNLLDQHT  929
             QNT NI NT+ALETFTRTMISSDREMRSLLRSRASIDIAAKT EKPV+CS  V+LLDQHT
Sbjct  541   QNTVNIHNTEALETFTRTMISSDREMRSLLRSRASIDIAAKTLEKPVICSERVSLLDQHT  720

Query  930   SGVDFTVKQVQ  962
              GVDFTV +++
Sbjct  721   QGVDFTVAEIE  753
```

Tobacco streak virus movement protein gene, complete cds
Sequence ID: MK307506.1Length: 873Number of Matches: 12
Range 1: 1 to 750GenBank Next Match Previous Match

Alignment statistics for match #1

Score	Expect	Identities	Positives	Gaps	Frame
492 bits(1068)	1e-139	198/250(79%)	231/250(92%)	0/250(0%)	+3/+1

```
Query  210   MALTPSYRALTFSADDESSLEKSVSDALSGCVELNMGLRRCAAFPATNTEAFLCELTTKE  389
             MAL P+ +ALTFSADDE+SLEK++++ALSG VELNMGLRRCAAFPA NT AFLCELTTKE
Sbjct  1     MALVPTMKALTFSADDETSLEKAITEALSGSVELNMGLRRCAAFPAVNTGAFLCELTTKE  180

Query  390   TKTILGKFADKVRGRVFVDHAVIHMMYIPVILSTTHAVAELKIRNMATGDELYGGTKVNL  569
             TK+ +GKF+DKVRGR FVDHAVIH++Y+PVIL TT+A++ELK++N+ATGDELYGGTKV+L
Sbjct  181   TKSFIGKFSDKVRGRTFVDHAVIHLLYLPVILKTTYAISELKLKNLATGDELYGGTKVDL  360

Query  570   NEAFILTMTWPRSLFADAVHAHKGLYLGGTVNCATSVPKGCNIGMWYPMWSEKVSNKQLY  749
             ++AFILTMTWPRSLFA+AVHAH+GLYLGGTV+CA+SVP    IGMWYPMWSEKVSNKQLY
Sbjct  361   SKAFILTMTWPRSLFAEAVHAHRGLYLGGTVSCASSVPSNAKIGMWYPMWSEKVSNKQLY  540

Query  750   QNTTNIVNTKALETFTRTMISSDREMRSLLRSRASIDIAAKTAEKPVLCSSHVNLLDQHT  929
             QNT NI +T+ALETFTRTMISSDREMRSLLRSRASIDIAA+T EKPV+CS  V+LLDQHT
Sbjct  541   QNTVNIHSTEALETFTRTMISSDREMRSLLRSRASIDIAARTPEKPVICSERVSLLDQHT  720

Query  930   SGVDFTVKQV  959
              GVDFTV ++
Sbjct  721   QGVDFTVSEI  750
```

Figure S2. Alignment of the amino-acid sequences of the PV-0309 PrVI isolate to closely related TSV isolates
TBLASTX search of the RNA sequence of PRVI DSMZ PV-0309 strain (OL584350) showed high identity in the amino-acid sequence of the movement protein encoded by TSV dahlia strain (LC030107) or by a TSV strain infecting summer squash in Georgia, U.S.A.
