# Supplementary material for: Clematis vitalba Is a Natural Host of the Novel Ilarvirus, Prunus Virus I
Source: Viruses. 2023 Sep 20;15(9):1964. doi: 10.3390/v15091964 (PMC10536899; doi:10.3390/v15091964)
Supplement: Supplementary file 1 [file viruses-15-01964-s001.zip › Salamon et al_Supplementary Material S1.pdf]

viruses

Supplementary Material S1 to

Clematis vitalba is a natural host of a novel ilarvirus, Prunus virus I

Pal Salamon[1], Zsuzsanna Nagyné-Galbács[2], Emese Demian[2], Adam Achs[3], Peter Alaxin[3,4], Lukáš Predajňa[3], Evans Duah Agyemang[5], Francesco Desiderio[2], Andras Takacs[5], Wulf Menzel[6], Dijana Škorić[7], Miroslav Glasa[3,4], Éva Varallyay[2]*

*Correspondence: varallyay.eva@uni-mate.hu

Bioinformatic analysis of the small RNA HTS

Small RNA sequencing revealed 15,370,590 reads 14,931,734 reads remained after trimming, representing 2,439,290 non-redundant reads. Size distribution of the sequenced reads showed peaks at 21, 22 and 24nt confirming that they are products of DICER enzymes.

Library code	Sequenced reads	Trimmed reads all (containing redundants)	Non-redundant reads	Number of contigs
209_Clematis	15370590	14931734	2439290	18754

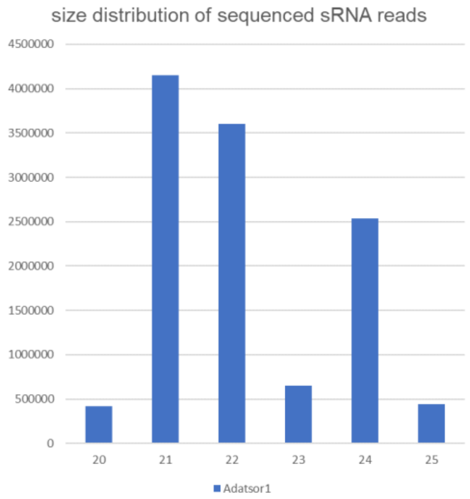

From these reads 18,754 contigs could be built. BLAST search of these contigs (carried out in early 2021) showed that 108 contigs could be mapped to the reference genomes of both three RNAs of several different ilarviruses. Moreover, 76 other contigs could be mapped to different badnaviruses, not more than two for any of them suggesting a presence of a badnavirus (these results are not discussed in details here). Mapping of the reads to the reference genomes of TSV, strawberry necrotic shock virus (SNSV), american plum line pattern virus (APLPV), parietaria mottle virus (PMV), citrus variegation virus (CVG), blackberry chlorotic ringspot virus (BCRV), ageratum latent virus (AgLV), privet ringspot virus (PRV), cape gooseberry ilarvirus 1 (CGIV1) showed that the number of mapped reads varied from 17-1667 read per million reads (RPM), and showed 17,5-51,7% coverage of the viral RNAs.

viruses

MDPI

Detailed results of the bioinformatics analysis of small RNA HTS of different libraries

	PrVI	MW579753	MW579754	MW579755
		RNA1	RNA2	RNA3
PrVI hit	number of contigs	885	792	518
	number of non-redundant reads	184852	112709	273927
	number of redundant reads	14612	11399	16679
	RPM	981	765	1119
	coverage of the viral genome (%)	97.3%	96.7%	97.1%

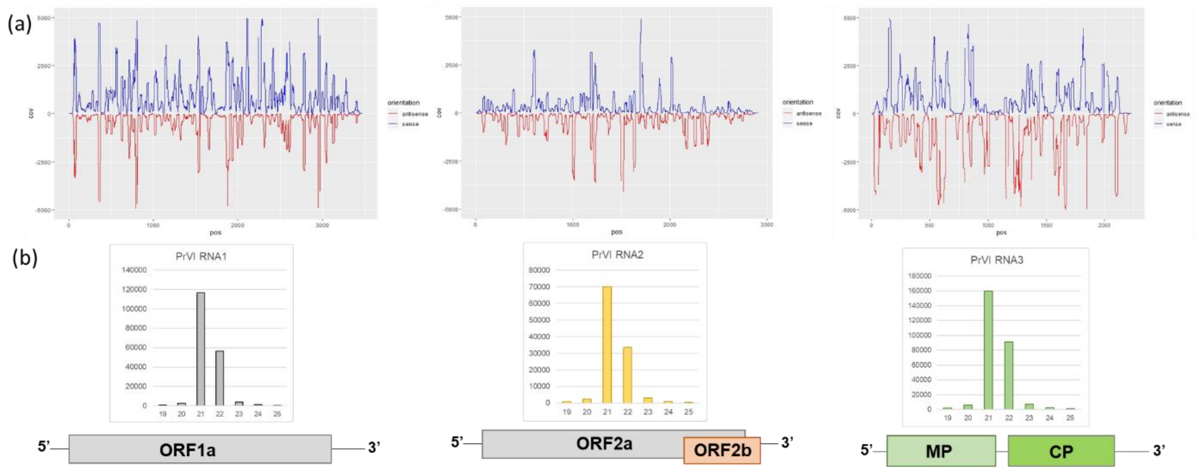

Profiles of PrVI-derived sRNAs. (a) distribution of the PrVI-derived sRNAs on the RNA1, RNA2 and RNA3. Sense and antisense sequence orientations are indicated by red and blue colors, respectively. (b) Size distribution of PrVI-derived sRNAs based on their origin. The representation of the corresponding genomic RNA can be found at the bottom of the Figure.
